# Psychometric properties of modified MOS social support survey 5-item (MSSS-5-item) among Iranian older adults

**DOI:** 10.1186/s12877-021-02353-0

**Published:** 2021-07-02

**Authors:** Maryam Bakhshandeh Bavarsad, Mahshid Foroughan, Nasibeh Zanjari, Zahra Jorjoran Shushtari, Gholamreza Ghaedamini Harouni

**Affiliations:** 1 Department of Aging, University of Social Welfare and Rehabilitation Sciences, Tehran, Iran; 2 Iranian Research center on Aging, Department of Aging, University of Social Welfare and Rehabilitation Sciences, Tehran, Iran; 3 Iranian Research Center of Aging, University of Social Welfare and Rehabilitation Sciences, Tehran, Iran; 4 Social Determinants of Health Research Center, University of Social Welfare and Rehabilitation Sciences, Tehran, Iran; 5 Social Welfare Management Research Center, University of Social Welfare and Rehabilitation Sciences, Tehran, Iran

**Keywords:** Social support, Older adult, Elderly, Psychometrics, Reliability, Validity, MSSS-5-item

## Abstract

**Background:**

Social support is a key factor in public health. Since the precise evaluation of it is critical, the current study has been developed to evaluate the psychometric properties of the MOS-SSS questionnaire’s abbreviated form (MSSS-5-item) among the Iranian older adults.

**Methods:**

This cross-sectional and methodological study was conducted on 420 community older adults (age ≥ 60) through random multi-stage sampling. The questionnaire was first translated into Persian through the Forward & Backward method based on WHO guidelines. Next, the validity of scales was investigated by calculating face validity, content validity, Known-group validity, explanatory factor analysis, and confirmatory factor analysis indices. The reliability of the questionnaire was calculated by internal consistency, test-retest, and absolute reliability. Moreover, the scalability of the questionnaire was checked through the Mokken scale analysis. The software packages SPSS version 22, AMOS version 22, and R (Mokken package) were employed to analyze the data.

**Results:**

the face validity was conducted using interviews with older adults and gathering the specialists’ opinions. Then, the items were grammatically and lexically corrected accordingly. The CVI index of the overall scale was 0.94, and for every single item above 0.89. The results of the independent t-test showed that the current questionnaire well distinguished between the older adults who do and do not feel lonely (*p* < 0.001). Two components were recognized according to the explanatory factor analysis. They together explained 67.78% of the total variance of the questionnaire. The CFA showed that the two-factor model had acceptable fit indices. The questionnaire had desirable internal consistency (α = 0.78), stability (ICC = 0.98), and absolute reliability (SEM = 0.56, MDC = 1.57). Furthermore, the Mokken scale proved that MSSS-5-item was a strong scale (H = 0.51, se = 0.03).

**Conclusion:**

The present study results showed that the MSSS-5-item questionnaire had suitable validity and reliability to be used among Iranian older adults.

## Background

Nowadays, there is a lot of evidence that shows social support has various short- and long-term effects on individuals’ physical and mental health and wellbeing due to its beneficial impacts (either directly or by stress buffer effect). Various studies point out the role of social support in health, wellbeing, and mitigating negative effects of stressful conditions and feeling lonely (loneliness) in older adults [1–9]. As a vital factor in people’s health, social support highlights the social aspect of humans [10]. Different resources such as friends, family, and society can offer social support whenever the person needs it [11, 12]. Generally, social support has three components: 1) the source of support, 2) satisfaction with the support, and 3) the type of support [12]. It also has four types: emotional support consisting of love, sympathy, care, and understanding; instrumental support, which provides material needs such as food; informational support involving information or suggestions for getting along with difficulties and hardships; appraisal support, which provides information facilitating self-assessment or affirming the person’s appropriateness of behavior or performance [12–14].

Perceived social support evaluates the individual’s perception of support, regardless of having received the support or not [15]. The perceived social support plays a crucial role in older adults’ lives; as people grow old, its importance increases progressively [16]. So, reasonably, social support has been greatly emphasized in the comprehensive geriatric assessment [11]. On the other hand, the shortage of social support is considered a modifiable risk factor that can be compensated through different interventions [11, 17, 18]. However, precise measurement is necessary before one can intervene in social support status.

Generally, social support is a meta-construct concept that lacks a unique definition and measurement method [18]. Various measuring scales have been designed due to the broad concept of social support, diverse components, and lack of a unique definition [13]. Some of these scales are used for the older adults’ population, including Norbeck Social Support Questionnaire (NSSQ) [19], interview schedule for social interaction [20], Social Support Questionnaire [21], and Multidimensional Scale of Perceived Social Support [22].

The Medical Outcomes Study Social Support Survey (MOS-SSS) is a widely used scale in assessing social support [23]. It is a 20-item scale with four components, developed by Sherbourne and Stewart in 1991, and was first validated and confirmed among 2987 patients with chronic disease [13]. The validity of this questionnaire has been confirmed on both sick [11, 17, 24–26] and healthy populations in various studies, too [23, 27–30]. Also, this questionnaire has been translated into other languages (such as Portuguese, Brazilian, Malaysian, Arabic, French, Chinese, Spanish, and Persian), and its psychometric properties have been evaluated in several studies, which have shown its proper characteristics [17, 24–27, 30, 31]. A review study conducted by Nazari et al. to assess the psychometric properties of the perceived social support scales indicated that the MOS-SSS survey was the finest questionnaire for use among older adults [18]. Another advantage of this questionnaire is having short and comprehensible items [11, 28]. The lack of items with reverse scoring is another positive feature of the MOSS-SSS survey, which reduces the errors in responses of individuals with lower education [28]. Since the original version of this questionnaire may exhaust the examinee due to a large number of items, several studies have applied the abbreviated forms of 8 items [11, 17, 29, 32], 6 items [23], 5 items [33–39], and 4 items [28] of this questionnaire. The abbreviated forms of this questionnaire also benefit from desirable psychometric features. Also, they are more suitable to be used among older adults because of fewer items, particularly in cases where several scales are to be used in a study [29]. The MSSS-5-item, an abbreviated version of the MOS-SSS, was designed by Ritvo et al. [40]. Ritvo et al. applied the MSSS-5-item in the Multiple Sclerosis Quality of Life Inventory (MSQLI) (α = 0.88) [40]. It also had good reliability in the older adults suffering from Multiple Sclerosis (α = 0.77) [33].

Based on our investigations, psychometric evaluation of the Modified MOS Social Support Survey 5-item has not been conducted so far. This questionnaire is suitable for use in the elderly population due to its briefness and comprehensibility; thus, the current study was developed to evaluate psychometric properties of the MSSS 5-item questionnaire among the Iranian older adults.

## Methods

### Research population and setting

This cross-sectional study was conducted on community-dwelling older adults who lived in Tehran, Iran. Tehran is Iran’s capital, with nearly 12 million people of various ethnicities, subcultures and socio-economic levels [41].

### Translation process

After receiving permission from the original developer of the questionnaire to translate and validate the questionnaire in Iran, the Forward & Backward method based on the WHO guidelines was used as follows [42]. First, the questionnaire was translated from English to Persian by two independent translators familiar with medical terms and fluent in English. Two translated versions were then compared in a session by the research team, and the translators and the discrepancies were resolved. Then, the translated Persian version was back-translated into English by two other translators who were not familiar with the original questionnaire to see whether items of the Persian version could transfer the purpose of the original one. Finally, the research team compared the backward translated version with the original version to see whether there is sufficient similarity or not. The original questionnaire can be reached by this link [40].

### Face validity

Qualitative face validity has been conducted through face to face interview with ten older adults. They were asked to evaluate the difficulty, relevancy and ambiguity of items.

The convenience sampling method was used to select older participants. The first author did all interviews, and the participants’ comments were assessed by the research team and used to correct the items.

### Content validity

The qualitative content validity was used for assessing the items according to grammar, wording, scaling, clarity and simplicity. 19 specialists consisting of gerontologists, geriatricians, geriatric nurses, psychologists, and sociologists with a PhD degree were asked to provide their comments for editing and revising the statements. Quantitative content analysis was applied at the next step by calculating the content validity index (CVI). CVI indices assess the relevancy of the items, which is considered the only index to evaluate CVI based on Polit et al. [43, 44].

In regard to the content validity index assessment for each item (I-CVI), initially, a 4-Point Likert scale (1 = not relevant, 2 = item needs major revision, 3 = relevant but needs minor revision, and 4 = completely relevant) was answered by several experts. Then, the content validity index was calculated by dividing the number of experts giving a rating of ‘3’ or ‘4’ to each statement by the total number of experts [45–47]. An I-CVI score over 0.79 was considered adequate [46]. Scale-CVI was calculated based on S-CVI/Avg, where the sum of I-CVI divided by their numbers [45]. Acceptable S-CVI/Avg based on Polit et al. opinion is ≥0.9 [44].

It is essential to evaluate chance agreement when there are several raters. So the present study used the modified Kappa statistic (K*), which designed by Polit et al. K* > 0.74 is considered as excellent [44] and calculated by the following equation [46, 48]:

$$ pc=\left[\frac{N!}{A!\left(N-A\right)!}\right]\ast {0.5}^N $$

$$ \mathrm{K}\ast =\frac{\mathrm{I}-\mathrm{CVI}-\mathrm{pc}}{1-\mathrm{pc}} $$

### Construct validity

In the present study, 420 eligible older adults were selected. Based on the rule of thumb, the sample size for factor analysis is categorized as follows: 50 = very poor, 100 = poor, 200 = fair, 300 = good, 500 = very good, and 1000 = excellent [49]. Eligibility criteria were age ≥ 60 years, with the ability to communicate, and adequate cognitive functioning based on abbreviated AMT test. The samples were randomly divided into two groups (210 subjects for exploratory factor analysis and 210 subjects for confirmatory factor analysis).

Samples were selected by a multi-stage cluster sampling method. At first, 22 districts of Tehran were classified into five groups in terms of socio-economic development levels from developed areas to underdeveloped (very poor) areas [50]. One district in each cluster and then two regions in each district were selected randomly. The sample size in each district was determined based on the proportion of its population to the total population. Kaiser-Meyer-Olkin (KMO) and Bartlett’s test of sphericity were used to evaluate Sample appropriateness. Bartlett’s test of sphericity should be statistically significant, and KMO values should be > 0.5 [51]. The pencil-and-paper instruments were used to collect the data by trained interviewers who could help if older adults had a problem filling the questionnaires.

### Explanatory and confirmatory factor analysis (EFA & CFA)

The components of MSSS-5-item were extracted by the EFA. The number of components was determined based on Kaiser’s criterion (eigenvalue > 1.0) and the Scree plot. Different extraction and rotation methods were used for better interpretation of extracted components. The factor loading of 0.35 and greater was necessary to retain an item in each component based on the following formula [52]: CV (critical value for accepting a factor loading) = 5.152/(N^− 2^) ^1/2^.

Confirmatory factor analysis was used to evaluate the goodness of fit indices based on maximum likelihood. The following indices and acceptable values were used in the present study; absolute fit indices including root mean score error of approximation (RMSEA< 0.08), goodness of fit index (GFI > 0.9), minimum discrepancy function divided by degrees of freedom (CMIN/DF < 3), standardized root mean square residual (SRMR< 0.1), comparative fit indices including comparative fit index (CFI > 0.9), normal fit index (NFI > 0.9) and parsimonious fit indices including adjusted goodness of fit index (AGFI> 0.8) [53].

### Known-group validity

Regarding the known-group validity, the individuals are divided into two groups according to the characteristic measured by the instrument: extremely high and extremely low. There should be a significant difference between the mean scores of the two group in order to claim to construct validity [54].

Several studies have confirmed that there is a significant negative relationship between social support and loneliness so that the older adults who feel lonely have less perceived social support [4, 5, 55, 56]. Loneliness was evaluated by a single question in which the respondents express their feeling of loneliness (1 = never to 5 = almost all of the time) [57]. Then, according to the responses, the participants were categorized into two groups; 1) feel lonely (respondents who were feeling loneliness sometimes to almost all the time) and 2) not feel lonely (respondents who were feeling loneliness never or rarely). An independent t-test was used to determine whether the means of social support were significantly different between the two groups.

### Convergent validity

This study used the SF8™ health survey to evaluate convergent validity. The SF8™ is an abbreviated SF 36 health survey consisting of two dimensions; physical component summary (PCS) and mental component summary (MCS). Both components have scores between 0 and 100, and higher scores show a better health status [58]. The Pearson correlation coefficient test was used to assess the correlation between social support and health status.

### Scalability of the questionnaire

Mokken scale analysis was used to explore the scalability of the items and analyze the item quality of the questionnaire [59]. The item scalability coefficient (Hi) of each item was calculated to determine the extent of discrimination power of the items. Furthermore, the Hi value of the item expresses the item’s quality, given the other items in the scale. Based on Molenaar and Sijtsma (2000) suggestion, Hi values ≤0.3 considered as a weak discrimination power, so the items should remain on a scale that has Hi > 0.3 [60]. The quality of the scale and scale homogeneity were evaluated by Loevinger’s H coefficients value. Scale quality has been categorized according to Sijtsma, K., & Molenaar, I. W. (2002) recommendation as follows: a scale with 0.3 ≤ H < 0.4 is considered as a weak scale; between 0.4 ≤ H < 0.5 a moderate scale and an H > 0.5 a strong scale [61].

### Reliability

The reliability of the MSSS-5-item was evaluated via stability, internal consistency and absolute reliability. Internal consistency was assessed by two indicators, Cronbach’s alpha and values above 0.7 were considered acceptable [62] and McDonald omega coefficients [63].

Stability was evaluated by the Test-retest technique, in which 30 older adults completed the questionnaire twice within a 2 weeks interval, then intra-class correlation coefficient (ICC) was computed. ICC was calculated using two-way mixed effects and absolute agreement at a 95% confidence interval. Intra-class correlation coefficient values above 0.75 were considered acceptable [64].

The absolute reliability can be calculated by two indexes: The standard error of measurement (SEM), Minimal detectable change (MDC). SEM represents to what extent the difference in measurement within different tests is because of a measurement error or reality [46]. Therefore, a lower amount of SEM indicates higher reliability [48].

On the other hand, MDC (also known as smallest detectable change (SDC) or smallest real difference) is defined as the smallest actual change, which is not because of the measurement error but because of real change. MDC is calculated through the SEM, and it is used for the interpretation of changes in scores [65]. SEM and MDC were calculated using the following equations [46, 48]:

$$ SEM=\mathrm{SD}\sqrt{1- ICC} $$

$$ MDC= SEM\times \sqrt{2}\times 1.96 $$

### Ceiling and floor effects

Ceiling and floor effects occur when more than 15% of participants choose responses at the higher and lower end of the scale, respectively, and show that the content validity is inappropriate. The ceiling and floor effects were calculated as the percentage for all data [66].

### Statistical data analysis

The SPSS version 22, R software (Mokken package), and the AMOS22 were used for data analysis. The following descriptive and analytical indices were used in the present study; Cronbach’s alpha, McDonald omega coefficients, Independent t-test, intra-class correlation coefficient, Pearson correlation coefficient, and Exploratory Factor Analysis and Confirmatory Factor Analysis.

## Result

### Face validity

A total of 10 older adults between the ages of 60–76 were interviewed. Roughly 30% of the participants were illiterate, 40 and 30% had a diploma and bachelor degree, respectively. Fifty percent of the sample was female, and all of them were married. No item was changed in the process of face validity.

### Content validity

In the qualitative content validity step, two items were revised based on given comments regarding grammar and wording. Modified Kappa statistic (K*) and I-CVI for all of the items were above.89. The average content validity index (S-CVI/Ave) was 0.94 for the total questionnaire.

### Construct validity

#### Demographic characteristics

A total of 420 older adults with a mean age of 69.03 ± 7.61 years (range of 60–93 years) recruited in this study. The most of participants were married (78.6%). The mean number of chronic diseases was 2.03 ± 1.26. Other demographic characteristics were summarized in Table 1 for total samples and EFA and CFA groups.

**Table 1:** Demographic Characteristics of the Participants

Variables		Total sample	CFA group	EFA group
		n (percent)	n (percent)	n (percent)
Gender	Male	224 (53.3)	107 (51)	117 (55.7)
	Female	196 (46.7)	103 (49)	93 (44.3)
Education	<High school	210 (50)	106 (50.5)	104 (49.5)
	≥High school	210 (50)	104 (49.5)	106 (50.5)
Marriage status	Single	9 (2.1)	5 (2.4)	4 (1.9)
	Married	330 (78.6)	159 (75.7)	171 (81.4)
	Widowed	70 (16.7)	41 (19.5)	29 (13.8)
	Divorced	11 (2.6)	5 (2.4)	6 (2.9)
Socio-economic Status	Developed	124 (29.5)	58 (27.6)	66 (31.4)
	Moderate developed	58 (13.8)	31 (14.8)	27 (12.9)
	Relatively developed	115 (27.4)	56 (26.7)	59 (28.1)
	less developed	62 (14.8)	35 (16.7)	27 (12.9)
	Under developed	61 (14.5)	30 (14.3)	31 (14.8)

#### Exploratory and confirmatory factor analysis

The results of Kaiser-Meyer-Olkin (KMO = 0.66) and Bartlett’s test of sphericity (*χ*^2^ = 542.98, df = 10, *P* < 0.001) indicated sampling adequacy. A set of exploratory factor analyses were conducted on the 5-item questionnaire. The best model, which was chosen by the Maximum Likelihood and Varimax rotation, included two components. The two components together explained 67.78% of the variance of MSSS-5-item (Table 2).

**Table 2:** Factor analysis using Maximum Likelihood and H_i_ coefficient

Components	Items	Hi (Se)	Factor loading	Variance %	Eigenvalue
Instrumental subscale (component1)	How often is someone available To take you to the doctor if you need to go?	0.551 (0.031)	0.896	36.38	1.81
	To prepare your meals if you are unable to do it yourself?	0.566 (0.029)	0.979		
Emotional subscale (component2)	To have a good time with?	0.525 (0.036)	0.859	31.4	1.57
	To hug you?	0.473 (0.036)	0.66		
	To understand your problems?	0.421 (0.041)	0.573		

**Fig. 1:**
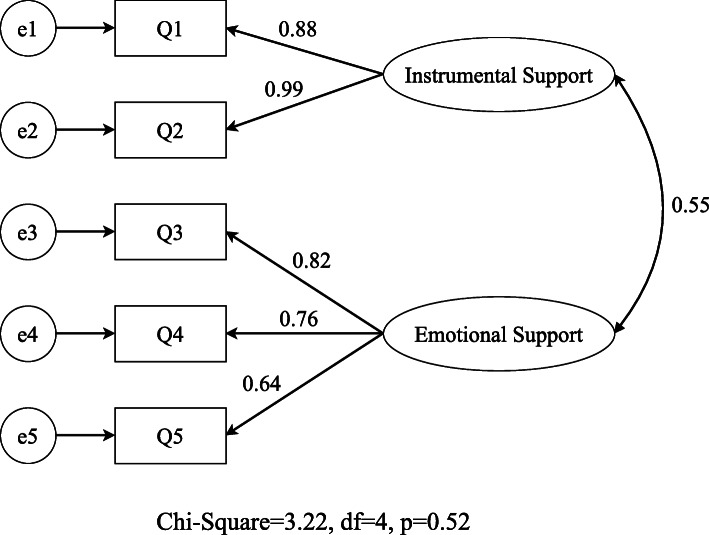
Confirmartory Factor Analysis of MSSS-5 items

Confirmatory factor analysis indicated that the final model extracted from the EFA had a good fit for the data (Fig. 1). Chi-squared test (X^2^ = 3.22, df = 4, *p* = 0.52) and absolute fit indices, comparative fit indices and parsimonious fit indices revealed that there is a good fit to the data RMSEA = 0.0001, GFI = 0.99, CMIN/DF = 0.8, SRMR = 0.02, CFI = 1.000, NFI = 0.99, AGFI = 0.97.

#### Known-group validity

The result of the independent t-test showed that there is a significant difference (*p* < 0.001) between the perceived social support of older adults who feel lonely (18.04 ± 4.57) and those who do not feel lonely (20.47 ± 3.24).

#### Convergent validity

The result of the Pearson correlation coefficient test showed that there was a significant positive correlation between the emotional, social support and both physical component (r = 0.13, *P* value< 0.05) and mental component of SF8™ (r = 0.36, *p* value< 0.001). Also, there was a significant negative correlation between instrumental support and physical component (r = − 0.18, *p* value< 0.001). Also, there was a negative correlation between instrumental support and physical health component; however, it was not significant.

#### Scalability and unidimensionality of the questionnaire

According to the Mokken scale analysis, the MSSS-5-item is a strong scale (H = 0.51, se = 0.03). Item scalability coefficient (H_i_) of each item was larger than 0.3, so all of the five items had a good discrimination power, and all of the items remained in scale. (Table 2). To test the unidimensionality of the scale, three assumptions/conditions required; a) positive item pair coefficients (H_ij_ > 0), b) H_j_ > .30, and c) H > .30 [67]. Our result showed that MSSS-5-item is scalable and unidimensional.

#### Reliability

Cronbach’s alpha for the total MSSS-5-item was 0.78, and McDonald’s Omega was 0.81. The reliability values based on the Mokken scale analysis were 0.78 and 0.81 for Cronbach’s alpha and Lambda, respectively. Moderate to high reliability was also found for each of the two subscales: Emotional support (alpha = 0.73); Instrumental support (alpha = 0.94).

The test-retest, intraclass correlation coefficient (ICC), was used to calculate the stability of the questionnaire. ICC for the total questionnaire was 0.98 (95% CI =0.96 to 0.99). ICC for the subscales; emotional support and instrumental support were 0.97 (95% CI =0.94–0.98) and 0.98 (95% CI =0.95–0.99), respectively.

Absolute reliability was assessed by two indicators, and the SEM and the MDC were 0.56 and 1.57, respectively. The ceiling and floor effects for the MSSS-5-item were 0 and 8.3%, respectively, that both were less than 15%.

## Discussion

The present study results showed that the abbreviated 5-item MOS-SSS questionnaire had a suitable validity and reliability for use in the older adults’ population living in Iran. Other studies have also been developed for psychometric evaluation of the abbreviated versions of this questionnaire; they examine the 4-, 6-, 8-, and 12-item versions in various populations and have obtained similar results [11, 17, 23, 28, 29]. It should be noted that the items vary in the different short forms due to the different approaches of the authors. In the study by Gomez-Campelo et al., Moser et al., and Togari et al., the first four items were selected in two emotional and instrumental components [11, 17, 29]. However, in the current study and the study by Holden et al. and Gjesfeld et al., the items were selected based on the highest level of factor loading and correlation with each component [23, 28].

The MSSS 5-item questionnaire is part of the Multiple sclerosis quality of life inventory survey developed by Ritvo et al. [40]. In the study of Ritvo et al., in order to construct the abbreviated version of social support, items from the MOS-SSS questionnaire that had the highest correlation with the total score of the scales were selected. Five items were selected from all four components of the MOS-SSS questionnaire, and an overall score was considered for it. Although the MSSS-5-item questionnaire created by Ritvo et al. does not have any components, the results from the explanatory factor analysis in this study showed that the scale consisted of two components, which overall explained 67.78% of the total variance. The extracted factors include emotional support (3 items) and instrumental support (2 items). The extracted factors in the present study are similar to the 8-item short forms in Moser et al. and Togari et al. [11, 29]. However, only one component has been reported for the 4- and 6-item versions [23, 28]. Gomez-Campelo et al.’s study used an 8-item form and showed that the explanatory factor analysis results in the two separate populations of men and women showed only one component [17]. This can be considered as the strength of the MSSS-5-item, which despite the small number of items, examines two types of social support. Moreover, another advantage of this questionnaire is the approach used for selecting items, compared to short 8-item versions.

One of the main reasons that makes it difficult to compare the present study results with other abbreviated versions of the MOS-SSS survey is the different populations under study. In the study by Gomez-Campelo et al., the studied population was 18- to 55-year-old Spanish individuals referred to the outpatient centers [17]. Gjesfeld et al.’s study focused on a group of American mothers with children under treatment [28]. The study by Holden et al. has also been done on Australian women of 28–58 years old [23]. Due to the specific population in each study, the results cannot totally be generalized. The study by Togari et al. has investigated 25 to 74 year-old people living in Japan [29]. In their study, explanatory factor analysis was performed on two age groups of below and over 50. Although Togari et al.’s study was conducted on a large number of individuals, what prevents their results from generalization to the elderly population is the age cutoff point (50 years old). According to the definition by the World Health Organization (WHO), the start of the old age is considered 60 and is respectively categorized as follows: young old (60–74), old old (75–84), oldest old (85+) [68]. Since the age range in that study was 25–74 years, it can be concluded that the studied population only included the young old. The study by Moser et al. was the only research showing that the 8-item form of social support survey had the desirable properties of psychometric evaluation in the elderly population over 65 years old [11]. Their study had a limitation by only investigating women. In the current study, however, the psychometric evaluation of the MSSS-5-item was specifically done on the older adults (the age range of 60–93 years old).

Regarding the investigation of the reliability of studies, various methods were used; for example, to reach an internal consistency, both Cronbach’s alpha and McDonald’s Omega were computed. Although Cronbach’s alpha is considered as a common indicator in investigating the internal consistency, McDonald’s Omega is also recommended when explanatory factor analysis is done. This indicator eliminates the shortcomings of the Chi-Square test, such as the number of questions and reverse-scores. The results show that the MSSS-5-item has a proper internal consistency based on both Chi-Square and McDonald’s Omega indicators. The value of Cronbach’s alpha in the present study (alpha = 0.78) matches the results of Dilorenzo et al.’s study, which applied the same questionnaire on the older adults with MS disease (alpha = 0.77) [33]. However, in the abbreviated 8-item questionnaire with similar sample size, Cronbach’s alpha in women and men over 50 years old was reported to be above 0.93 [29]. This contrast may be due to the difference in the number of items in the two questionnaires, as an increase in the number of items usually leads to an increase in the degree of correlation between them, thereby increasing Cronbach’s alpha. In addition to the internal consistency, two more stability and absolute reliability indices were also examined in this study, while only internal consistency is reported in other abbreviated versions [11, 17, 23, 28, 29]. These indices had desirable values in the current study indicative of good reliability of the scale.

The current study showed that the MSSS-5-item well differentiated between the older adults feeling and not feeling lonely. This result shows the suitable discriminant validity of this questionnaire. Also, in the study by Moser et al., the abbreviated 8-item questionnaire indicated a significant difference between women who felt lonely and those who did not [11]. This study showed that there is a negative correlation between instrumental support and health status that is approved with other studies [69, 70]. To summarize, people with deteriorated health gain more instrumental support [29]. Also, a positive correlation between emotional support and health confirmed that having more supporting resources help people to maintain a good health condition [29].

The results of the Mokken scale analysis showed that the present questionnaire was scalable and unidimensional with strong scalability (H > 0.5). According to the results, all items in this questionnaire measure a latent variable and can be a powerful indicator for measuring social support.

The present study was conducted in Tehran as the research setting. As the capital of Iran, Tehran possesses a population with high diversity in ethnicity and socio-economic levels. This study tried to benefit from maximum diversity in sampling by applying the random sampling method and dividing the city into different zones, based on socio-economic status, from underdeveloped to fully developed areas. However, as a research limitation, this questionnaire has been validated in the community-dwelling older adults; so, a reevaluation is needed for use in other populations. Moreover, the results of this study cannot be generalized to the elderly suffering from cognitive disorders.

## Conclusion

The results of the current study indicated that the MSSS 5-item has desirable validity and reliability among Iranian community older adults. In addition, the two-factor model has acceptable fit indices. The abbreviated 5-item social support questionnaire also has good scalability. The smaller number of items in this questionnaire minimizes the participants’ burden compared with the original version with 19 items. This is a major benefit of the present scale for use in older adults. The small number of psychometric studies on the abbreviated versions of the MOS-SSS questionnaire indicates the need for designing further studies. In addition, the lack of a unified abbreviated form prevents comparison between different studies, which is a limitation that should be addressed in the future.

## Data Availability

All data that support the findings of this study are not publicly available due to participants’ confidentiality.

## Abbreviations

MOS-SSS: Medical Outcomes Study Social Support Survey
MSSS-5-item: Modified MOS Social Support Survey 5-item
MSQLI: Multiple Sclerosis Quality of Life Inventory
CVI: Content validity index
KMO: Kaiser-Meyer-Olkin
EFA: Explanatory factor analysis
CFA: Confirmatory factor analysis
RMSEA: Root mean score error of approximation
GFI: Goodness of fit index
CMIN/DF: Minimum discrepancy function divided by degrees of freedom
SRMR: Standardized root mean square residual
CFI: Comparative fit index
NFI: Normal fit index
AGFI: Adjusted goodness of fit index
Hi: Item scalability coefficient
ICC: Intra-class correlation coefficient
SEM: Standard error measurement
DC_95_: Minimal detectable change
CI: Confidence interval
